# Natural cases of polyarthritis associated with feline calicivirus infection in cats

**DOI:** 10.1007/s11259-022-09933-4

**Published:** 2022-05-05

**Authors:** Andrea Balboni, Ranieri Verin, Isotta Buldrini, Silvia Zamagni, Maria Morini, Alessia Terrusi, Laura Gallina, Lorenza Urbani, Francesco Dondi, Mara Battilani

**Affiliations:** 1 Department of Veterinary Medical Sciences, Alma Mater Studiorum-University of Bologna, Ozzano dell’Emilia, Bologna, Italy; 2 Department of Comparative Biomedicine and Food Science, University of Padova, Legnaro, Padua, Italy

**Keywords:** Cat, Feline calicivirus, Lameness, Limping, Polyarthritis, Synovial fluid

## Abstract

The limping syndrome is occasionally reported during acute feline calicivirus (FCV) infections or as consequence of vaccination. In this retrospective study, three clinical cases of lameness in household cats naturally infected by FCV were described and phylogeny of the virus were investigated by analysing the hypervariable E region of the ORF2 viral gene. Cats were diagnosed with polyarthritis and FCV RNA or antigens were detected in symptomatic joints. One cat, euthanized for ethical reasons, underwent a complete post-mortem examination and was subjected to histopathological and immunohistochemical investigations. No phylogenetic subgrouping were evident for the sequenced FCV. Histopathology of the euthanized cat revealed diffuse fibrinous synovitis and osteoarthritis eight months after the onset of lameness and the first detection of FCV RNA, supporting the hypothesis of a persistent infection. FCV was demonstrated by immunohistochemistry in synoviocytes and fibroblasts of the synovial membranes. This study provides new data on the occurrence of polyarthritis in FCV-infected cats, demonstrates by immunohistochemistry the presence of FCV in the synovial membranes of a cat with persistent polyarthritis and supports the absence of correlation between limping syndrome and phylogenetic subgrouping of viruses.

**Supplementary Information:**

The online version contains supplementary material available at 10.1007/s11259-022-09933-4.

## Introduction

Feline calicivirus (FCV, family Caliciviridae, genus *Vesivirus*) is usually responsible for acute or subacute clinical signs in cats involving the upper respiratory tract with presence of lingual or oral ulcerations (Caringella et al. 2019; Radford et al. 2007). Several other clinical presentations were reported in FCV-infected cats including virulent systemic disease (Battilani et al. 2013) and feline chronic gingivostomatitis (Thomas et al. 2017). Limping syndrome is the less investigated clinical form associated to FCV infection. It is characterized by polyarthritis, fever and lameness, associated or not with upper respiratory tract disease (URTD). Between the 80 s and 90 s some studies reported cases of limping syndrome during acute FCV infections or as consequence of vaccination (Dawson et al. 1993; Levy and Marsh 1992; Pedersen et al. 1983). Experimental infections of specific pathogen-free (SPF) cats with FCV strains isolated from cats showing limping syndrome or vaccine strains showed controversial identification of the virus in the affected joints. Pedersen and collaborators (1983) reproduced lameness by inoculating the virus oro-nasally but no virus or viral antigens were detected in the joints. Bennet and collaborators (1989) reported the presence of viral antigens in the joints of cats experimentally infected both with field and vaccine strains but no animals showed lameness. Differently, Dawson and collaborators (1994) isolated the virus from both normal and affected joints of SPF cats natural exposed to a field FCV strain or inoculated intra-articularly with a vaccine strain. The virus was also isolated in a newly vaccinated household kitten from an affected joint that showed severe mononuclear inflammation in the synovial fluid (Levy and Marsh 1992).

Although Pedersen (1992) suggested that lameness should be considered a typical, but underestimated, presentation of FCV infection, currently no data are available on the actual occurrence of polyarthritis in FCV-infected cats. In this study, clinical cases of polyarthritis in household cats naturally infected by FCV were described and phylogeny of the FCV identified were investigated.

## Materials and methods

This was a retrospective study carried out at the Veterinary University Hospital (VUH) of the Department of Veterinary Medical Sciences, University of Bologna. Cats referred to the VUH in 2017–2019 reporting lameness were included in the study if polyarthritis was diagnosed and FCV RNA or antigens were detected in symptomatic joints. Polyarthritis was diagnosed in cats that showed systemic signs of inflammation (lethargy, anorexia and fever) and reluctance to walk associated with at least two swollen and painful joints (Lemetayer and Taylor 2014). The diagnosis was confirmed by synovial fluid cytology compatible with suppurative inflammation in two or more joints (Lemetayer and Taylor 2014), see Online Resource 1. No cats were sampled exclusively for the purposes of this study. Only samples taken for diagnostic purposes following owner’s consent were used. All analyses were carried out at the time of cats’ care at the VUH on fresh non-stored biological samples, with the exception of viral genome sequencing and immunohistochemistry (IHC) which were carried out at the time of the study on RNA extracts stored at -80 °C and organ samples stored in paraffin blocks, respectively. Signalment data, vaccination status and clinicopathological findings of each cat included in the study were retrieved from medical records.

The presence of FCV RNA was investigated in synovial fluid samples (taken from two or more symptomatic joints) and also in conjunctival, oropharyngeal and nasal pooled swabs. Arthrocentesis was performed following the indications of Lemetayer and Taylor (2014). Viral RNA was extracted from synovial fluid (pooled samples) and from swabs, using the QIAamp Viral RNA Mini Kit (Qiagen, Germany). FCV RNA detection was carried out at the time of extraction using a SYBR Green real-time reverse transcription PCR (RT-qPCR) assay (Helps et al. 2002), as reported in Online Resource 2. The reactions were carried out using the EXPRESS One-Step SYBR GreenER Kit (Thermo Fisher Scientific, USA) and the StepOnePlus Real-Time PCR System (Thermo Fisher Scientific, USA). Serial tenfold dilutions of a plasmid (pCR4 plasmid, Life Technologies, USA) containing from 1 × 10^0^ to 1 × 10^7^ copies of the target sequence for microliter were used as external standards for the construction of the standard curve. Melting experiments were carried out with a continuous increment from 60 to 95 °C and the specific melting temperature (Tm) was about 81 °C. The limit of detection (LOD) of the assay, assessed by testing serial tenfold dilutions of the recombinant plasmid, was 1 copy of target amplicon/µL. The RNA samples and standards were tested in duplicate. A no template control underwent analysis simultaneously. The specimens were considered positive if the amplification fluorescence curve increased exponentially, the Tm was specific and the mean of the target copy number obtained from the replicates was greater than the LOD.

The 3’ fragment of the ORF2, containing the hypervariable E region, was amplified from identified FCV using the primers FW4 (CCTGATGGTTGGCCAGACAC) and FR4 (GTACCCTTTGCTCAAGAATTTTGT) previously reported (Battilani et al. 2013), see Online Resource3. Reverse transcription and amplification were carried out using the SuperScript IV VILO MasterMix (Thermo Fisher Scientific, USA) and the Phusion Hot Start II High-Fidelity DNA Polymerase (Thermo Fisher Scientific, USA), respectively. Amplicons of the expected size (about 950 nucleotides, from nucleotide 6562 to nucleotide 7509 of the FCV strain F9 M86379) were sequenced by Sanger method using both forward and reverse primers. The nucleotide sequences obtained were assembled and aligned with 65 FCV reference sequences (Online Resource4) using ClustalW and translated into amino acid sequences using BioEdit 7.2.5. (Hall 1999). Phylogenetic relationships were evaluated using MEGA11 version 11.0.10 (Tamura et al. 2021).

A cat euthanized for ethical reasons underwent a complete post-mortem examination within 24 h of death and relevant organs, including synovial membranes, were collected and fixed in 10% buffered formalin, embedded in paraffin, sectioned at four-µm and stained with haematoxylin and eosin for histopathological investigations. The synovial membranes sampled were also immunohistochemically labeled using a mouse monoclonal antibody anti-FCV capsid protein (clone CV8-1A) provided by Custom Monoclonals International (West Sacramento, USA), see Online Resource5. As IHC positive control, a formalin fixed and paraffin embedded cell pellet obtained from a PCR confirmed FCV-infected cell culture was used. Negative controls were obtained by replacing the primary antibody with a non-reacting polyclonal antibody. Synovial fluid was also sampled during necropsy and directly tested (without storage at -80 °C) for the presence of FCV RNA by RT-qPCR.

## Results

Three cats showing clinical and clinicopathological signs supportive of polyarthritis were included in the study. Clinicopathological findings are reported in Online Resource6, the three cats had leucocytosis, neutrophilia and increased serum amyloid A. FCV RNA was detected only in the synovial fluid (pooled samples from left carpus and right knee) with a quantity of 1.3 × 10^2^ copies of target amplicon/µL of extract (copies/µL) for Cat1 (ID: 1101/2017), both in synovial fluid (pooled samples from left carpus, left and right elbows and left knee) and swabs for Cat2 (ID: 1466/2017) with a quantity of 5.8 × 10^4^ copies/µL and 9.7 × 10^4^ copies/µL respectively and only in swab samples with a quantity of 7.6 × 10^1^ copies/µL for Cat3 (ID: 1072/2018). In Cat3, FCV was demonstrated by IHC in the synovial membranes. The three cats were domestic short-hair neutered males from the province of Bologna and lived indoors with outdoor access. Cat1 was nine-year-old and showed clinical signs of polyarthritis with generalized lymphoadenopathy but no fever. Cat2 was 11-month-old and showed clinical manifestation of polyarthritis associated to prosencephalic signs. Cat3 was seven-year-old and was affected by polyarthritis associated to chronic dermatopathy (hyperkeratosis, desquamation and diffuse folliculitis); the cat had recurrent lameness for eight months from the first detection of FCV RNA in oropharyngeal swab in 2018 and was euthanized in 2019, despite medical treatment, for ethical reasons. The three cats had no signs of upper respiratory tract disease. Cat2 and Cat3 were regularly vaccinated against feline panleukopenia virus, feline herpesvirus and FCV infections, whereas, for Cat1 this data was not available.

**Fig. 1:**
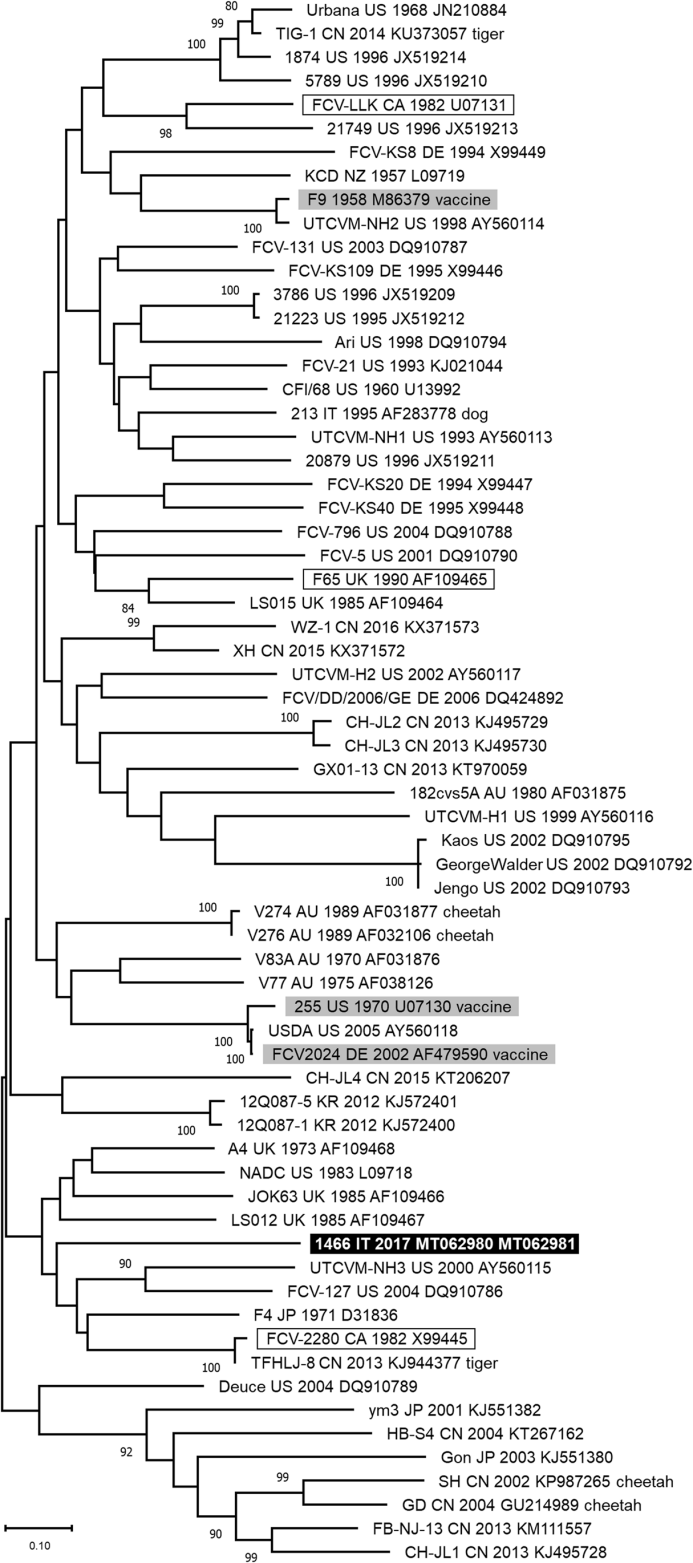
Unrooted phylogenetic tree constructed with nucleotide sequences generated in this study and FCV reference sequences available from GenBank (Online Resource4). The tree was constructed on the nucleotide alignment of the 3’ fragment of ORF2, comprised between nucleotides 6604 and 7329 of the reference strain F9 M86379. The best-fit model of nucleotide substitution was determined using the Find Best DNA/Protein Model function implemented in MEGA 11. Generalised time reversible model with gamma distribution and invariant sites resulted optimal for the sequence data. Phylogenetic trees were constructed using Maximum Likelihood method and bootstrap values were determined by 1000 replicates to assess the confidence level of each branch pattern. Bootstrap values greater than 80% are indicated on the respective branches. Identification of the sequences undergoes the following nomenclature: strain, country (AU: Australia, CA: Canada, CN: China, DE: Germany, IT: Italy, JP: Japan, KR: South Korea, NZ: New Zeeland, UK: United Kingdom, US: United States of America), collection date (or date of database submission), GenBank accession number and host species other than the cat. In bold: nucleotide sequence generated in this study. Highlighted in grey: vaccine reference strains. Framed: reference strains identified in cats showing lameness

A fragment of 873 nucleotides of FCV genome, containing the last 717 nucleotides of the ORF2, was sequenced from the two different biological matrices of Cat2: pooled synovial fluids (MT062980) and pooled swabs (MT062981). For the other two cats, no specific PCR product was obtained from the amplification of the ORF2 gene and consequently no viral nucleotide sequences were obtained. The two nucleotide sequences were identical and showed a nucleotide identity ranging from 68.4% to 77.5% with all the reference sequences analysed. Phylogeny did not allow to cluster the FCV sequences on geographical, temporal or clinical basis (Fig. 1).

**Fig. 2:**
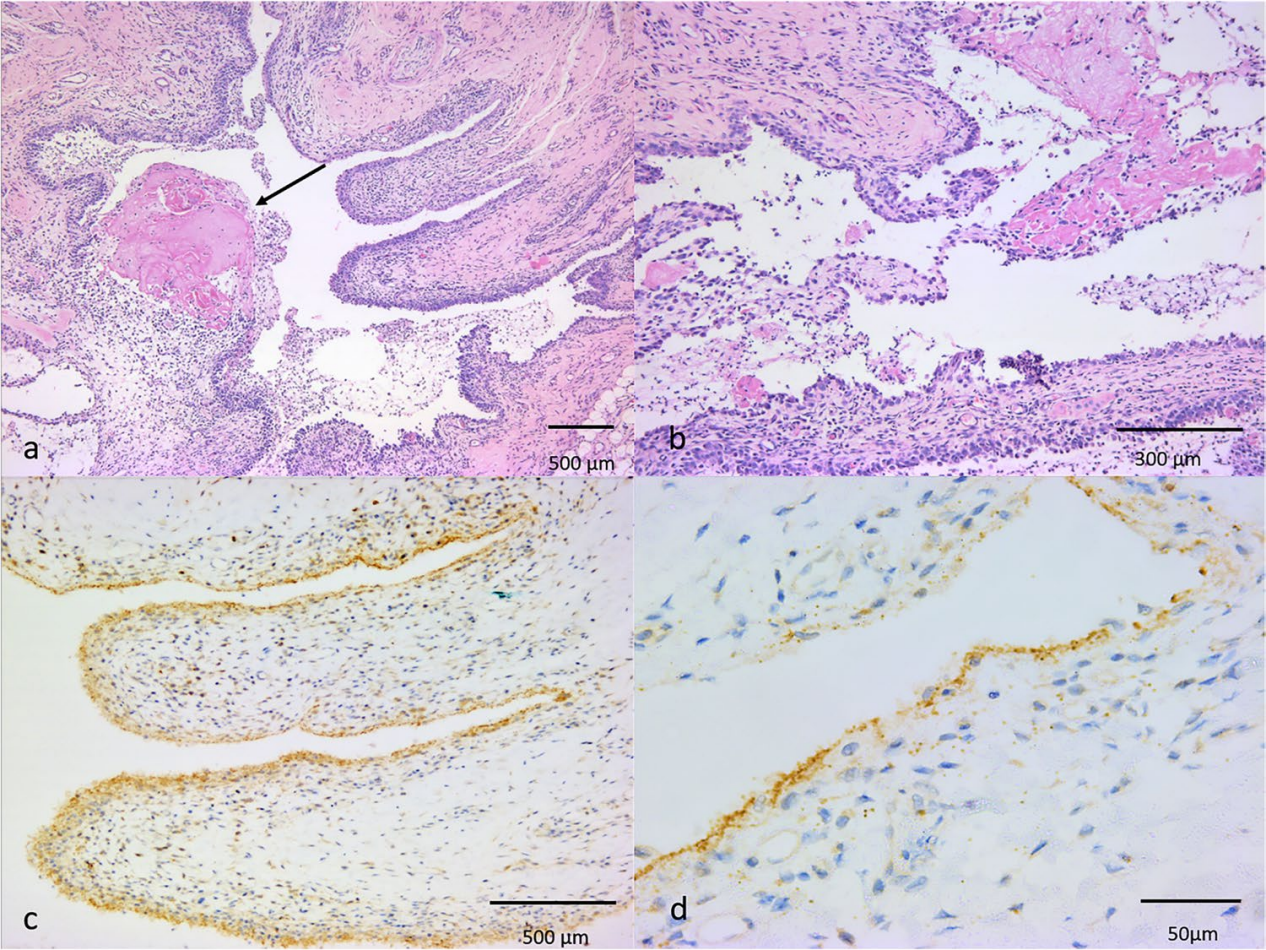
Joints of Cat3 (lab ID: 1072/2018). **a** Histology of left carpus showing moderate diffuse hyperplasia of synoviocytes constituting the intimal layer, fibrin deposition (arrow) and inflammatory infiltrates. Scale bar = 500 µm. Haematoxylin and eosin. **b** Histology of left tarsus showing numerous viable and degenerate neutrophils admixed with fibrin. Scale bar = 300 µm. Haematoxylin and eosin. **c** Hyperplastic synoviocytes in the intimal layer in the left carpus showing positive cytoplasmic immunostaining for FCV antigens. Scale bar = 500 µm. Immunohistochemistry (IHC). **d** Left tarsus, positive cytoplasmic immunostaining for FCV antigens mainly observed in synoviocytes of the intimal layer and less frequently in scattered fibroblasts in the subintimal layer. Scale bar = 50 µm. IHC

Post-mortem examination of Cat3 revealed moderate pallor of skin and mucous membranes, and the joints (right and left carpus and right and left tarsus) showed diffuse thickening and synovial proliferation, oedema and multifocal haemorrhages with an excess of synovial fluid. The liver was diffusely moderately congested. No other significant gross abnormalities were observed in the other organs except of the dermatopathy. Histopathology showed moderate to severe (depending on the joint examined), sub-acute, diffuse fibrinous synovitis with moderate to severe folding and hyperplasia of the synovial membranes. Viable and degenerate neutrophils and macrophages were also detected in all the examined joints in variable distribution depending on the joint examined associated, in two of the examined locations, to small areas of coagulative necrosis (Table 1 and Fig. 2). A focally extensive area of osteoarthritis was also observed in the left stifle. The remaining organs only showed variable degree of congestion more severely evident in the lung with scattered thrombosed small to medium calibre pulmonary vessels. IHC demonstrated FCV antigen in the cytoplasm of synoviocytes lining and constituting the intimal layer and less frequently in scattered fibroblasts in the sub-intimal layer of all the synovial membranes tested with different degree of positive immunostaining (Table 1 and Fig. 2). Synovial fluid samples obtained at post-mortem examination tested negative to FCV RNA.

**Table 1:** Main histopathological lesions and associated scoring at different locations including immunolabel score for FCV antigen obtained by means of immunohistochemistry (IHC) in Cat3 (lab ID: 1072/2018)

	L carpus	R carpus	L knee	R knee	L tarsus	R tarsus
Synovial hyperplasia	+ + +	+ +	+	+	+ + +	+
Fibrin	+ + +	+ +	+	+	+ + +	+
Inflammation	+ + +	+	+	+	+ + +	+
Necrosis	+	–	–	–	+	–
IHC	+ + + +	+ +	+ +	+	+ +	+ +

Histopathological score: – = no lesions; +  = minimal; +  +  = mild; +  +  +  = moderate; +  +  +  +  = severe; +  +  +  +  +  = complete loss of structure

IHC score (distribution of FCV antigen, as determined by immunohistochemistry): – = negative; +  = occasional presence of immunolabelled cells; +  +  = small number of cells; +  +  +  = moderate; +  +  +  +  = numerous; +  +  +  +  +  = widespread immunolabelling

*IHC* immunohistochemistry; *L* left; *R* right

## Discussion

In this study, the detection of FCV RNA or antigens in symptomatic joints is strongly indicative of the presence of polyarthritis in FCV-infected cats. The three cats included in this study had synovial fluid cytology consistent with suppurative inflammation (> 1000 nucleated cells/mL with > 10% of neutrophils, Lemetayer and Taylor 2014) and showed haematological and serum chemistry findings indicative of inflammation, consistent with polyarthritis. To the authors' knowledge, only one previous study reported partial clinicopathological findings of an FCV-infected kitten with arthritis (Levy and Marsh 1992) which, unlike the cats included in this study, exhibited leukopenia with a mild left shift and toxic neutrophils, probably resulting from acute viremia or recent vaccination. The detection of FCV antigens in the synovial membranes of a cat (Cat3) showing severe polyarthritis and lameness, without detection of viral RNA in the synovial fluid, supports the hypothesis that FCV can be found as immune-complex aggregates in the joints of infected cats (Bennett et al. 1989) or act as a trigger of immune-mediated response involving the joints as reported for other aetiologies (Lemetayer and Taylor 2014). Since the synovial fluid of this cat was tested for FCV RNA immediately after sampling (without undergoing freezing and thawing), the negative result obtained cannot be related to the viral RNA degradation. Rather, it can be assumed that in Cat3 the virus (and its RNA) was harboured in a compartment not directly accessible by arthrocentesis. Furthermore, the synovial membranes of Cat3 were analysed eight months after the onset of lameness and the first detection of FCV RNA in the upper respiratory tract, suggesting a persistent infection, frequently reported for FCV (Coyne et al. 2006; Wardley 1976). This finding is also in contrast to a transient lameness with possible recover after few days from clinical onset (Pedersen et al. 1983).

The hypervariable E region of the ORF2 gene, was sequenced for one FCV identified in this study. For the other two cats, it was not possible to obtain FCV genome sequences, possibly as a consequence of the low amount of viral RNA detected in the samples tested. No phylogenetic subgrouping were evident for the FCV identified in Cat2 as previously reported (Glenn et al. 1999; Hou et al. 2016; Radford et al. 1997). The viral nucleotide sequences obtained from both synovial fluid and swabs samples of this cat were identical, therefore a coinfection with two viruses with different tissue tropism is excluded. The low nucleotide identity detected among the virus sequenced and all the reference sequences analysed is in accordance with the current literature (Pesavento et al. 2008). Indeed, numerous studies reported high genetic distance between epidemiologically unrelated FCV, regardless of the clinical presentation in infected cats (Coyne et al. 2012; Glenn et al. 1999; Hou et al. 2016; Radford et al. 1997). Although at least two cats included in this study were regularly vaccinated against FCV infection and lameness was reported in kittens after the first vaccination (Dawson et al. 1993), no evidence was found that FCV vaccine strains contributed to the pathology as the FCV sequences obtained from Cat2 revealed no genetic relationship with the vaccine strains included in the phylogenetic tree. Further studies are warranted to confirm association of polyarthritis with FCV vaccination.

There are some limitations in this study. A small number of cats was included, so prospective studies, with a larger number of cats and stringent inclusion criteria, would be needed to accurately assess the frequency of polyarthritis in FCV-infected cats. Furthermore, the detection of FCV RNA in synovial fluid samples, even if strongly indicative, does not prove the cause-effect relationship between the infection and the onset of polyarthritis, therefore the presence of other concomitant and undiagnosed causes of polyarthritis, however unlikely, cannot be ruled out with absolute certainty in the two cats not subjected to IHC.

In conclusion, this study provides new data on the occurrence of polyarthritis in FCV-infected cats, demonstrates by IHC the presence of FCV in the synovial membranes of a cat with persistent polyarthritis and supports the absence of correlation between limping syndrome and phylogenetic subgrouping of viruses.

## Supplementary Information

Below is the link to the electronic supplementary material.

Supplementary file1 (PDF 266 kb)

Supplementary file2 (PDF 264 kb)

Supplementary file3 (PDF 262 kb)

Supplementary file4 (PDF 281 kb)

Supplementary file5 (PDF 260 kb)

Supplementary file6 (PDF 268 kb)

## Data Availability

The datasets generated and analysed during the current study are available in the International Nucleotide Sequence Database Collaboration (INSDC) repository (http://www.insdc.org/; ID: MT062980 and MT062981).

## References

[CR1] Battilani M, Vaccari F, Carelle MS, Morandi F, Benazzi C, Kipar A, Dondi F, Scagliarini A (2013). Virulent feline calicivirus disease in a shelter in Italy: a case description. Res Vet Sci.

[CR2] Bennett D, Gaskell RM, Mills A, Knowles J, Carter S, McArdle F (1989). Detection of feline calicivirus antigens in the joints of infected cats. Vet Rec.

[CR3] Caringella F, Elia G, Decaro N, Martella V, Lanave G, Varello K, Catella C, Diakoudi G, Carelli G, Colaianni ML, Bo S, Buonavoglia C (2019). Feline calicivirus infection in cats with virulent systemic disease, Italy. Res Vet Sci.

[CR4] Coyne KP, Christley RM, Pybus OG, Dawson S, Gaskell RM, Radford AD (2012). Large scale spatial and temporal genetic diversity of feline calicivirus. J Virol.

[CR5] Coyne KP, Dawson S, Radford AD, Cripps PJ, Porter CJ, McCracken CM, Gaskell RM (2006). Long-term analysis of feline calicivirus prevalence and viral shedding patterns in naturally infected colonies of domestic cats. Vet Microbiol.

[CR6] Dawson S, Bennett D, Carter SD, Bennett M, Meanger J, Turner PC, Carter MJ, Milton I, Gaskell RM (1994). Acute arthritis of cats associated with feline calicivirus infection. Res Vet Sci.

[CR7] Dawson S, McArdle F, Bennett D, Carter SD, Bennett M, Ryvar R, Gaskell RM (1993). Investigation of vaccine reactions and breakdowns after feline calicivirus vaccination. Vet Rec.

[CR8] Glenn M, Radford AD, Turner PC, Carter M, Lowery D, DeSilver DA, Meanger J, Baulch-Browne C, Bennett M, Gaskell RM (1999). Nucleotide sequence of UK and Australian isolates of feline calicivirus (FCV) and phylogenetic analysis of FCVs. Vet Microbiol.

[CR9] Hall TA (1999). BioEdit: a user-friendly biological sequence alignment editor and analysis program for Windows 95/98/NT. Nucl Acids Symp Ser.

[CR10] Helps C, Lait P, Tasker S, Harbour D (2002). Melting curve analysis of feline calicivirus isolates detected by real-time reverse transcription PCR. J Virol Methods.

[CR11] Hou J, Sánchez-Vizcaíno F, McGahie D, Lesbros C, Almeras T, Howarth D, O’Hara V, Dawson S, Radford AD (2016). European molecular epidemiology and strain diversity of feline calicivirus. Vet Rec.

[CR12] Lemetayer J, Taylor S (2014). Inflammatory joint disease in cats: diagnostic approach and treatment. J Feline Med Surg.

[CR13] Levy JK, Marsh A (1992). Isolation of calicivirus from the joint of a kitten with arthritis. J Am Vet Med Assoc.

[CR14] Pedersen NC (1992). Inflammatory oral cavity diseases of the cat. Vet Clin North Am Small Anim Pract.

[CR15] Pedersen NC, Ekman S, Laliberte L (1983). A transient febrile “limping” syndrome of kittens caused by two different strains of feline calicivirus. Feline Pract.

[CR16] Pesavento PA, Chang KO, Parker JS (2008). Molecular virology of feline calicivirus. Vet Clin North Am Small Anim Pract.

[CR17] Radford AD, Bennett M, McArdle F, Dawson S, Turner PC, Glenn MA, Gaskell RM (1997). The use of sequence analysis of a feline calicivirus (FCV) hypervariable region in the epidemiological investigation of FCV related disease and vaccine failures. Vaccine.

[CR18] Radford AD, Coyne KP, Dawson S, Porter CJ, Gaskell RM (2007). Feline calicivirus. Vet Res.

[CR19] Tamura K, Stecher G, Kumar S (2021). MEGA11: molecular evolutionary genetics analysis version 11. Mol Biol Evol.

[CR20] Thomas S, Lappin DF, Spears J, Bennett D, Nile C, Riggio MP (2017). Prevalence of feline calicivirus in cats with odontoclastic resorptive lesions and chronic gingivostomatitis. Res Vet Sci.

[CR21] Wardley RC (1976). Feline calicivirus carrier state. A study of the host/virus relationship. Arch Virol.

