# Supplementary material for: Natural cases of polyarthritis associated with feline calicivirus infection in cats
Source: Vet Res Commun. 2022 May 5;46(2):613–9. doi: 10.1007/s11259-022-09933-4 (PMC9165229; doi:10.1007/s11259-022-09933-4)
Supplement: Supplementary file 1 — Supplementary file1 (PDF 266 kb) [file 11259_2022_9933_MOESM1_ESM.pdf]

Veterinary Research Communications

Natural cases of polyarthritis associated with feline calicivirus infection in cats

Andrea Balboni, Ranieri Verin, Isotta Buldrini, Silvia Zamagni, Maria Morini, Alessia Terrusi, Laura Gallina, Lorenza Urbani, Francesco Dondi, Mara Battilani.

* Corresponding author:

Francesco Dondi

Department of Veterinary Medical Sciences, *Alma Mater Studiorum* – University of Bologna, Ozzano dell'Emilia (BO), Italy

E-mail address: f.dondi@unibo.it

Online Resource 1 Supplementary materials and methods: Findings of synovial fluid cytology examination compatible with inflammatory polyarthritis

The diagnosis of inflammatory polyarthritis was confirmed by synovial fluid cytology compatible with suppurative inflammation in two or more joints (Lemetayer and Taylor 2014; Stone 2017). Synovial fluid samples were analysed at the time of cats' care and considered to have suppurative inflammation if they showed an increase in total nucleated cell counts: > 2 cells/400X objective lens (Barger 2016) or > 1000 cells/mL if assessed using an automated cell counter (Pacchiana et al. 2004), and neutrophils represented > 10% of total nucleated cells (Clements et al. 2004; Rondeau et al. 2005) and exhibit non-degenerated morphology (Darren et al. 2021).

References

Barger AM (2016) Muscoloskeletal System. In: Rose E, Meyer D (eds) Canine and feline cytology, a color atlas and interpretation guide, Third Edn. Saunders Elsevier, St. Louis, MO, USA, pp 353-368

Clements DN, Gear RN, Tattersall J, Carmichael S, Bennett D (2004) Type I immune-mediated polyarthritis in dogs: 39 cases (1997-2002). J Am Vet Med Assoc 224:1323-1327. https://doi. org/10.2460/javma.2004.224.1323

Darren Wood R, Gibson T (2021). In: Sharkey LC, Radin MJ, Seelig D (eds) Synovyal fluid analysis of the dog and cat, veterinary cytology, First Edn. Wiley-Blackwell Hoboken, NJ, USA, pp 727-733

Pacchiana PD, Gilley RS, Wallace LJ, Hayden DW, Feeney DA, Jessen CR, Aird B (2004). Absolute and relative cell counts for synovial fluid from clinically normal shoulder and stifle joints in cats. J Am Vet Med Assoc 225:1866-1870. https://doi.org/10.2460/javma.2004.225.1866

Rondeau MP, Walton RM, Bissett S, Drobatz KJ, Washabau RJ (2005) Suppurative, nonseptic polyarthropathy in dogs. J Vet Intern Med 19:654-662. https://doi.org/10.1892/0891-6640(2005)19[654:snpid]2.0.co;2

Stone M (2017) Immune-mediated polyarthritis and other polyarthritides. In: Ettinger SJ, Feldman EC, Côté E (eds) Textbook of veterinary internal medicine, Eighth Edn. Saunders Elsevier, St. Louis, MO, USA, pp 861-865
