# Supplementary material for: Natural cases of polyarthritis associated with feline calicivirus infection in cats
Source: Vet Res Commun. 2022 May 5;46(2):613–9. doi: 10.1007/s11259-022-09933-4 (PMC9165229; doi:10.1007/s11259-022-09933-4)
Supplement: Supplementary file 2 — Supplementary file2 (PDF 264 kb) [file 11259_2022_9933_MOESM2_ESM.pdf]

Veterinary Research Communications

Natural cases of polyarthritis associated with feline calicivirus infection in cats

Andrea Balboni, Ranieri Verin, Isotta Buldrini, Silvia Zamagni, Maria Morini, Alessia Terrusi, Laura Gallina, Lorenza Urbani, Francesco Dondi, Mara Battilani.

* Corresponding author:

Francesco Dondi

Department of Veterinary Medical Sciences, *Alma Mater Studiorum* – University of Bologna, Ozzano dell'Emilia (BO), Italy

E-mail address: f.dondi@unibo.it

Online Resource 2 Supplementary materials and methods: One-step real-time reverse transcriptase-PCR (RT-qPCR) performed for FCV RNA detection

The presence of FCV RNA was investigated by using a SYBR Green one-tube real-time reverse transcriptase-PCR (RT-qPCR) targeting a fragment of 83 nucleotides in the ORF1 region, with the primers forward (5′- TAA TTC GGT GTT TGA TTT GGC CTG GGC T -3′) and reverse (5′- CAT ATG CGG CTC TGA TGG CTT GAA ACT G -3′) (Helps et al., 2002). The reaction was performed using the EXPRESS One-Step SYBR GreenER Kit (Thermo Fisher Scientific, USA) according to the manufacturer's instruction, in a total volume of 20 µL, and the StepOnePlus Real-Time PCR System (Thermo Fisher Scientific, USA). The thermal cycling consisted of a reverse transcription step at 50 °C for 5 min, followed by 95 °C for 2 min and 40 cycles of 95 °C for 15 sec and 60 °C for 50 sec. Melting experiment for the evaluation of the specificity of the reaction was performed after the last extension step by a continuous increment from 60°C to 95°C and specific melting temperature (Tm) was about 81 °C. FCV RNA copies number determination was carried out by absolute quantification using the standard curve method. Serial 10-fold dilutions of a plasmid (pCR4 plasmid, Life Technologies, USA) containing from 1×10^0 to 1×10^7 copies of the FCV target sequence for microliter were used as external standards for the construction of the assay standard curve by plotting the plasmid copy number against the corresponding threshold cycle values. The limit of detection (LOD) of the reaction was determined based on the highest dilution of recombinant plasmid possible to amplify with good reproducibility and was found to be one copies/µL. The RNA samples and standards were repeated within each run in duplicate. A no template control, consisting of ultrapure water, underwent analysis simultaneously. Samples showing an exponential increase in the fluorescence curve, a target DNA amount greater than or equal to the LOD and a specific melting peak in both replicates were considered positive.
