# Supplementary material for: Natural cases of polyarthritis associated with feline calicivirus infection in cats
Source: Vet Res Commun. 2022 May 5;46(2):613–9. doi: 10.1007/s11259-022-09933-4 (PMC9165229; doi:10.1007/s11259-022-09933-4)
Supplement: Supplementary file 3 — Supplementary file3 (PDF 262 kb) [file 11259_2022_9933_MOESM3_ESM.pdf]

Veterinary Research Communications

Natural cases of polyarthritis associated with feline calicivirus infection in cats

Andrea Balboni, Ranieri Verin, Isotta Buldrini, Silvia Zamagni, Maria Morini, Alessia Terrusi, Laura Gallina, Lorenza Urbani, Francesco Dondi, Mara Battilani.

* Corresponding author:

Francesco Dondi

Department of Veterinary Medical Sciences, *Alma Mater Studiorum* – University of Bologna, Ozzano dell'Emilia (BO), Italy

E-mail address: f.dondi@unibo.it

Online Resource 3 Supplementary materials and methods: Two-step reverse transcription-PCR (RT-PCR) performed to amplify the 3’ fragment of the FCV ORF2

RNA extract stored at -80 °C was retrotranscribed to cDNA using the SuperScript IV VILO Master Mix (Thermo Fisher Scientific, USA) according to the manufacturer’s instruction. A no template control, consisting of ultrapure water, underwent analysis simultaneously. cDNA was stored at −20 °C until use. A fragment of about 950 nucleotides of ORF2 region (from nucleotide 6562 to nucleotide 7509 of the FCV strain F9 M86379) was amplified by using a qualitative PCR with the primers FW4 (CCTGATGGTTGGCCAGACAC) and FR4 (GTACCCTTTGCTCAAGAATTTTGT) previously reported (Battilani et al. 2013). The reaction was performed using the Phusion Hot Start II DNA Polymerase (Thermo Fisher Scientific, USA), containing a high-fidelity DNA polymerase, according to the manufacturer’s instruction, in a total volume of 50 μL. The thermal cycling consisted of 98 °C for 30 sec, 45 cycles of 98 °C for 15 sec, 58 °C for 60 sec and 72 °C for 45 sec, followed by a final elongation step at 72 °C for 10 min. The no template control amplified in the reverse transcription step and a no template control, consisting of ultrapure water, underwent analysis simultaneously. Five microlitres of each amplicon was separated by electrophoresis in a 2% (W/V) agarose gel stained with ethidium bromide in 1 X Tris-acetate ethylene diamine tetra-acetic acid (TAE) buffer, together with a GeneRuler 100 bp Plus DNA Ladder (Fermentas, USA), and visualised with ultraviolet (UV) light.
