# Supplementary material for: Natural cases of polyarthritis associated with feline calicivirus infection in cats
Source: Vet Res Commun. 2022 May 5;46(2):613–9. doi: 10.1007/s11259-022-09933-4 (PMC9165229; doi:10.1007/s11259-022-09933-4)
Supplement: Supplementary file 4 — Supplementary file4 (PDF 281 kb) [file 11259_2022_9933_MOESM4_ESM.pdf]

Veterinary Research Communications

Natural cases of polyarthritis associated with feline calicivirus infection in cats

Andrea Balboni, Ranieri Verin, Isotta Buldrini, Silvia Zamagni, Maria Morini, Alessia Terrusi, Laura Gallina, Lorenza Urbani, Francesco Dondi, Mara Battilani.

* Corresponding author:

Francesco Dondi

Department of Veterinary Medical Sciences, *Alma Mater Studiorum* – University of Bologna, Ozzano dell'Emilia (BO), Italy

E-mail address: f.dondi@unibo.it

Online Resource 4 Supplementary materials and methods: Feline calicivirus reference strains retrieved from GenBank (https://www.ncbi.nlm.nih.gov/genbank/) used in the molecular analysis

Strain	Origin	Year	GenBank ID	Host	Clinical signs and notes
**213**	IT	1995	AF283778	Dog	Diarrhoea
**255**	US	1970	U07130	Cat	Oral lesions and pneumonia - Vaccine strain
**1874**	US	1996	JX519214	Cat	URTD
**3786**	US	1996	JX519209	Cat	Asymptomatic
**5789**	US	1996	JX519210	Cat	Chronic stomatitis
**20879**	US	1996	JX519211	Cat	Chronic stomatitis
**21223**	US	1995	JX519212	Cat	Pneumonia
**21749**	US	1996	JX519213	Cat	Chronic stomatitis
**12Q087-1**	KR	2012	KJ572400	Cat	NA
**12Q087-5**	KR	2012	KJ572401	Cat	NA
**182cvs5A**	AU	1980	AF031875	Cat	URTD
**A4**	UK	1973	AF109468	Cat	URTD
**Ari**	US	1998	DQ910794	Cat	VS
**CFI/68**	US	1960	U13992	Cat	URTD
**CH-JL1**	CN	2013	KJ495728	Cat	Asymptomatic
**CH-JL2**	CN	2013	KJ495729	Cat	URTD
**CH-JL3**	CN	2013	KJ495730	Cat	URTD
**CH-JL4**	CN	2015	KT206207	Cat	NA
**Deuce**	US	2004	DQ910789	Cat	VS
**F4**	JP	1971	D31836	Cat	URTD
**F65**	UK	1990	AF109465	Cat	Lameness and stomatitis
**F9**	NA	1958	M86379	Cat	Vaccine strain
**FB-NJ-13**	CN	2013	KM111557	Cat	NA
**FCV/DD/2006/GE**	DE	2006	DQ424892	Cat	NA
**FCV-127**	US	2004	DQ910786	Cat	URTD
**FCV-131**	US	2003	DQ910787	Cat	URTD
**FCV2024**	DE	2002	AF479590	Cat	Vaccine strain

**FCV-21**	US	1993	KJ021044	Cat	Asymptomatic
**FCV-2280**	CA	1982	X99445	Cat	Lameness
**FCV-5**	US	2001	DQ910790	Cat	VS
**FCV-796**	US	2004	DQ910788	Cat	URTD
**FCV-KS109**	DE	1995	X99446	Cat	Chronic stomatitis
**FCV-KS20**	DE	1994	X99447	Cat	Chronic stomatitis
**FCV-KS40**	DE	1995	X99448	Cat	URTD
**FCV-KS8**	DE	1994	X99449	Cat	Acute stomatitis
**FCV-LLK**	CA	1982	U07131	Cat	Lameness
**GD**	CN	2004	GU214989	Cheetah	NA
**George Walder**	US	2002	DQ910792	Cat	VS
**Gon**	JP	2003	KJ551380	Cat	NA
**GX01-13**	CN	2013	KT970059	Cat	NA
**HB-S4**	CN	2004	KT267162	Cat	NA
**Jengo**	US	2002	DQ910793	Cat	VS
**JOK63**	UK	1985	AF109466	Cat	URTD and stomatitis
**Kaos**	US	2002	DQ910795	Cat	VS
**KCD**	NZ	1957	L09719	Cat	Asymptomatic
**LS012**	UK	1985	AF109467	Cat	Chronic stomatitis
**LS015**	UK	1985	AF109464	Cat	Chronic stomatitis
**NADC**	USA	1983	L09718	Cat	Asymptomatic
**SH**	CN	2002	KP987265	Cheetah	NA
**TFHLJ-8**	CN	2013	KJ944377	Tiger	NA
**TIG-1**	CN	2014	KU373057	Tiger	NA
**Urbana**	US	1968	JN210884	Cat	URTD
**USDA**	US	2005	AY560118	Cat	URTD
**UTCVM-H1**	US	1999	AY560116	Cat	VS
**UTCVM-H2**	US	2002	AY560117	Cat	VS
**UTCVM-NH1**	US	1993	AY560113	Cat	URTD
**UTCVM-NH2**	US	1998	AY560114	Cat	URTD
**UTCVM-NH3**	US	2000	AY560115	Cat	URTD

**V274**	AU	1989	AF031877	Cheetah	URTD and oral lesions
**V276**	AU	1989	AF032106	Cheetah	URTD and oral lesions
**V77**	AU	1975	AF038126	Cat	Oral lesions and abortion
**V83A**	AU	1970	AF031876	Cat	Sudden death and lung injury
**WZ-1**	CN	2016	KX371573	Cat	NA
**XH**	CN	2015	KX371572	Cat	NA
**ym3**	JP	2001	KJ551382	Cat	NA

AU = Australia; CA = Canada; CN = China; DE = Germany; IT = Italy; JP = Japan; KR = South Korea; NA = not available; NZ = New Zeeland; UK = United Kingdom; URTD = upper respiratory tract disease; US = United States of America; VS = virulent systemic.
