# Supplementary material for: Natural cases of polyarthritis associated with feline calicivirus infection in cats
Source: Vet Res Commun. 2022 May 5;46(2):613–9. doi: 10.1007/s11259-022-09933-4 (PMC9165229; doi:10.1007/s11259-022-09933-4)
Supplement: Supplementary file 5 — Supplementary file5 (PDF 260 kb) [file 11259_2022_9933_MOESM5_ESM.pdf]

Veterinary Research Communications

Natural cases of polyarthritis associated with feline calicivirus infection in cats

Andrea Balboni, Ranieri Verin, Isotta Buldrini, Silvia Zamagni, Maria Morini, Alessia Terrusi, Laura Gallina, Lorenza Urbani, Francesco Dondi, Mara Battilani.

* Corresponding author:

Francesco Dondi

Department of Veterinary Medical Sciences, *Alma Mater Studiorum* – University of Bologna, Ozzano dell'Emilia (BO), Italy

E-mail address: f.dondi@unibo.it

Online Resource 5 Supplementary materials and methods: FCV immunohistochemistry (IHC) in synovial membranes

The synovial membranes sampled from Cat3 (lab ID: 1072/2018) and stored in paraffin blocks were immunohistochemically stained using a mouse monoclonal antibody anti-FCV capsid protein (clone CV8-1A) provided by Custom Monoclonals International (USA). Sections were mounted on positively charged slides, deparaffinised in xylene, and placed in ethanol. Endogenous peroxidase activity was blocked with 0.3% hydrogen peroxide in methanol for 10 minutes followed by decreasing concentrations of ethanol and hydration in water. Antigen retrieval was achieved with proteinase K (Dako, Agilent, USA). Tissues then were rinsed in phosphate-buffered saline (PBS). The slides were incubated for one hour at room temperature in a humidified chamber applying 100 μL each of the primary monoclonal antibody diluted 1:100 in PBS. After primary antibody incubation, the slides were rinsed with tris-buffered saline with Tween 20 (TBST) (Dako, Agilent, USA). The slides were labelled with anti-mouse EnVision System with horseradish peroxidase (HRP) (Dako, Agilent, USA) for 30 min at room temperature and eventually rinsed with TBST. The chromogen (3-amino-9-ethylcarbazole - AEC substrate, Dako, Agilent, USA) was applied for 10 min before a final rinse and counterstaining with Papanicolaou's 1b Hematoxylin Solution (Merck, Sigma-Aldrich, USA), and coverslipped. As IHC positive control, a formalin fixed and paraffin embedded cell pellet obtained from a PCR confirmed FCV infected cell culture was used. Negative controls were obtained by replacing the primary antibody with a non-reacting polyclonal antibody.
