# Supplementary material for: Natural cases of polyarthritis associated with feline calicivirus infection in cats
Source: Vet Res Commun. 2022 May 5;46(2):613–9. doi: 10.1007/s11259-022-09933-4 (PMC9165229; doi:10.1007/s11259-022-09933-4)
Supplement: Supplementary file 6 — Supplementary file6 (PDF 268 kb) [file 11259_2022_9933_MOESM6_ESM.pdf]

Veterinary Research Communications

Natural cases of polyarthritis associated with feline calicivirus infection in cats

Andrea Balboni, Ranieri Verin, Isotta Buldrini, Silvia Zamagni, Maria Morini, Alessia Terrusi, Laura Gallina, Lorenza Urbani, Francesco Dondi, Mara Battilani.

* Corresponding author:

Francesco Dondi

Department of Veterinary Medical Sciences, *Alma Mater Studiorum* – University of Bologna, Ozzano dell'Emilia (BO), Italy

E-mail address: f.dondi@unibo.it

Online Resource 6 Supplementary results: Clinicopathological findings of the cats included in the study

**Variables**	**Cat1**	**Cat2**	**Cat3**	**Reference interval**
Haematology				
RBCs (cells/mm^3)	8,540,000	7,820,000	6,680,000	7,000,000–11,000,000
WBCs (cells/mm^3)	44,230	22,580	27,590	4800–14,930
Hb (g%)	11.8	11.1	8.7	10–16
Hct (g%)	35.3	32.2	27.1	32–48
MCV (fl)	41.3	41.1	40.6	36–55
MCH (pg)	13.8	14.2	13.1	12.3–16.2
MCHC (g%)	33.4	34.6	32.2	31–36
MPV (fl)	18.9	28.5	13.3	8–26
RDW (%)	16.4	14.3	15.8	13.17–17
Platelets (cells/mm^3)	155,000	87,000	461,000	150,000–500,000
Reticulocytes (cells/mm^3)	46,500	51,200	46,100	0–80,000
Neutrophils (cells/mm^3)	41,320	20,780	24,720	1600–10,000
Monocytes (cells/mm^3)	650	440	1000	0–650
Lymphocytes (cells/mm^3)	1690	1280	1670	900–5600
Basophils (cells/mm^3)	120	10	10	0–60
Eosinophil (cells/mm^3)	430	0	90	60–1470
Serum Chemistry				
ALT (U/l)	13	48	11	20–72
AST (U/l)	24	48	22	9–40
ALP (U/l)	4	6	9	0–140
GGT (U/l)	0.2	0.1	0.1	0–4
Total Bilirubin (mg/dl)	0.35	1.54	0.17	0–0.3
Cholesterol (mg/dl)	105	147	171	64–229
Glucose (mg/dl)	116	193	119	63–148
Albumin (g/dl)	2.12	3	2.67	2.6–4
Total Protein (g/dl)	6.41	7.04	6.8	6.5–8.8
A:G (g:g)	0.49	0.74	0.65	0.52–1.19

Creatinine (mg/dl)	1.11	0.9	0.67	0.8–1.8
Urea (mg/dl)	41.75	28.39	38.82	30–65
Phosphate (mg/dl)	5.65	5.63	5.47	2.5–6.2
Potassium (mEq/l)	4	3.8	4.8	3.4–5.1
Sodium (mEq/l)	150	144	153	145–155
Chloride (mEq/l)	117.7	106.5	120.4	110–123
Magnesium (mg/dl)	2.47	2.62	2.88	1.9–2.8
Total Calcium (mg/dl)	8.4	8.6	9.3	8.5–10.5
SAA (µg/dl)	121	199	82	0–10

CBC was carried out using an automated haematology analyser (ADVIA 2120; Siemens Healthcare Diagnostics). The haematology was completed with a microscopic blood smear examination using May-Grünwald Giemsa staining. Serum chemistry profile, including creatinine, urea, phosphate, total protein, albumin, the albumin to globulin ratio (A:G), alanine transaminase, aspartate transaminase, alkaline phosphatase, gamma(γ)-glutamyltransferase, total bilirubin, cholesterol, total calcium, sodium, potassium, chloride, glucose, and serum amyloid A (SAA) was determined. Serum chemical analysis was carried out using an automated analyser (AU480; Beckman Coulter-Olympus).

A:G = albumin to globulin ratio; ALP = alkaline phosphatase level; ALT = alanine aminotransferase; AST = aspartate aminotransferase; GGT = gamma(γ)-glutamyl transferase; Hb = hemoglobin; Hct = hematocrit value; MCH = mean corpuscular haemoglobin; MCHC = mean corpuscular haemoglobin concentration; MCV = mean corpuscular volume; MPV = mean platelet volume; RBCs = red blood cells; RDW = red cell distribution width; SAA = serum amyloid A; WBCs = white blood cells.
